# Switching of Pyruvate Kinase Isoform L to M2 Promotes Metabolic Reprogramming in Hepatocarcinogenesis

**DOI:** 10.1371/journal.pone.0115036

**Published:** 2014-12-26

**Authors:** Carmen Chak-Lui Wong, Sandy Leung-Kuen Au, Aki Pui-Wah Tse, Iris Ming-Jing Xu, Robin Kit-Ho Lai, David Kung-Chun Chiu, Larry Lai Wei, Dorothy Ngo-Yin Fan, Felice Ho-Ching Tsang, Regina Cheuk-Lam Lo, Chun-Ming Wong, Irene Oi-Lin Ng

**Affiliations:** 1 Department of Pathology, The University of Hong Kong, Hong Kong, HKSAR; 2 State Key Laboratory for Liver Research, The University of Hong Kong, Hong Kong, HKSAR; University of Nebraska Medical Center, United States of America

## Abstract

Hepatocellular carcinoma (HCC) is an aggressive tumor, with a high mortality rate due to late symptom presentation and frequent tumor recurrences and metastasis. It is also a rapidly growing tumor supported by different metabolic mechanisms; nevertheless, the biological and molecular mechanisms involved in the metabolic reprogramming in HCC are unclear. In this study, we found that pyruvate kinase M2 (PKM2) was frequently over-expressed in human HCCs and its over-expression was associated with aggressive clinicopathological features and poor prognosis of HCC patients. Furthermore, knockdown of PKM2 suppressed aerobic glycolysis and cell proliferation in HCC cell lines *in* *vitro*. Importantly, knockdown of PKM2 hampered HCC growth in both subcutaneous injection and orthotopic liver implantation models, and reduced lung metastasis *in* *vivo*. Of significance, PKM2 over-expression in human HCCs was associated with a down-regulation of a liver-specific microRNA, miR-122. We further showed that miR-122 interacted with the 3UTR of the PKM2 gene. Re-expression of miR-122 in HCC cell lines reduced PKM2 expression, decreased glucose uptake *in* *vitro*, and suppressed HCC tumor growth *in* *vivo*. Our clinical data and functional studies have revealed a novel biological mechanism involved in HCC metabolic reprogramming.

## Introduction

Hepatocellular carcinoma (HCC) is the sixth most prevalent cancer and the third most common fatal cancer worldwide [1]. Liver has many unique metabolic functions including gluconeogenesis, glycogen synthesis and storage, as well as blood glucose homeostasis. As HCC is highly proliferative, altered metabolic mechanisms are required to support its rapid demand for nutrients. Better understanding of the metabolic machinery of HCC is fundamental to devising better therapeutic strategies against HCC.

Normal differentiated cells metabolize glucose through oxidative phosphorylation in the presence of oxygen and through glycolysis in the absence of oxygen. Contrarily, despite a less energy-efficient metabolic route, cancer cells metabolize glucose through glycolysis even in the presence of oxygen and the phenomenon is called the Warburg Effect [2]. Increase of glucose uptake and production of lactate are typical signatures of the Warburg Effect [2]. Accumulating studies have shown that this preferential metabolic reprogramming to glycolysis channels glucose intermediates for the maximal biomolecule and anti-oxidant production [3].

Pyruvate kinases (PKs) are located at the pivotal position of glycolysis and catalyze the last step of glycolysis in which the phosphate group from phosphoenolypyruvate (PEP) is transferred to ADP, producing pyruvate and ATP. Pyruvate kinases (PKs) include 4 isoforms encoded by 2 paralogous genes, the *PKL* and *PKM* genes. The *PKL* gene generates PKL and PKR isoforms that are driven by different tissue-specific promoters [4]. The *PKM* gene is alternatively spliced by mutual exclusion of the 10^th^ and 9^th^ exons to generate PKM1 and PKM2, respectively [5]. PKL is abundantly expressed in normal liver and kidney; PKR is abundantly expressed in red blood cells; PKM1 is abundantly expressed in adult muscle, brain, bladder, and fibroblasts, whereas PKM2 is abundantly expressed in tissues during embryogenesis. PKM2 is also the predominant form that is over-expressed in multiple cancers including lung [6], colorectal [7], and gastric cancer [8]. Furthermore, PKM2 over-expression has been suggested to be associated with advanced stage and lymph node metastasis in colorectal cancer and associated with poor prognosis in gastric cancer [8]. Studies have shown that PKM2 is less glycolytically active than PKM1 [6], [9], therefore allowing the channeling of glucose intermediates upstream of pyruvates for antioxidant (NADPH), fatty acid, and nucleotide production [3]. Replacement of PKM2 by other PK isoform in lung cancer cell line markedly decreased glycolytic activity and suppressed tumor growth [6].

We herein report that PKM2, but not other PK isoforms, was over-expressed in human HCCs and its over-expression was associated with aggressive clinicopathological features and poor prognosis. Stable knockdown of PKM2 in multiple HCC cell lines decreased glucose uptake and lactate accumulation. Stable knockdown of PKM2 increased intracellular reactive oxygen species (ROS) level, which has been shown to be detrimental to cancer cells [10]. In addition, stable knockdown of PKM2 hampered HCC proliferation *in* *vitro* and tumorigenicity and metastasis *in* *vivo*. Intriguingly, we showed that PKM2 expression was regulated by the most abundant microRNA in the liver, miR-122. We found that miR-122 interacted with the 3UTR of PKM2 and suppressed PKM2 expression in human HCCs. Re-expression of miR-122 in HCC cell lines also decreased glucose uptake and lactate accumulation. Importantly, expression levels of PKM2 and miR-122 inversely correlated in human HCCs and non-tumorous liver tissues. Our results have revealed that the miR-122-mediated switch of PK isoforms drove HCC metabolic reprogramming.

## Materials and Methods

### Patient samples and cell lines

Human HCC and NT liver tissues were collected at Queen Mary Hospital, the University of Hong Kong during surgical resection. Samples were snap-frozen or fixed in formalin immediately. Use of human samples was approved by the Institutional Review Board of the University of Hong Kong/Hospital Authority Hong Kong West Cluster. Our institutional review board or ethics committee waived the need for consent. Human HCC cell lines, BEL-7402 and SMMC-7721, were gifts from Shanghai Institute of Cell Biology, Chinese Academy of Sciences. Metastatic human HCC cell line, MHCC-97L, was gift from Dr. Z.Y. Tang from Fudan University of Shanghai. HepG2 and PLC/PRF/5 were obtained from American Type Culture Collection (ATCC).

### Establishment of PKM2 knockdown and miR-122 over-expressing HCC cells

pLKO.1 plasmid carrying shRNA sequences against PKM2 (shPKM2-88: NM_182471.1-1070s1c1, shPKM2-94: NM_182471.1-1736s1c1), PKL (shPKL-39: NM_000298.4-1376s1c1, shPKL-94: NM_000298.4-221s1c1) and non-target control (NTC) were purchased from Sigma (St Louis, MO). pMIRNA1 plasmid carrying miR-122 precursor sequences was purchased from System Biosciences (Mountain View, CA). To generate HCC cells that stably express –NTC and –shPKM2 or –EV and -miR-122 precursor sequences, pLKO/pMIRNA1 plasmids and viral packaging plasmids (System Biosciences) were transfected into 293FT packaging cells. Viral particles were collected and infected into parental HCC cells with polybrene (Sigma). Puromycin was used to select stable transfectants.

### Luciferase reporter assay and transfection of miR-RNA precursors and locked nucleic acids (LNAs)

Wild-type and mutated 3UTR of PKM2 were inserted into pmirGLO Dual-luciferase miRNA target expression vector (Promega, Madison, WI). Sensor sequences, perfectly complementary to the miR-122, were included as positive control. A day after these constructs were transiently transfected into BEL-7402 cells by Lipofectamine 2000 (Life Technologies, Grand Island, NY), 5 pmol of miR-122 precursors (Life Technologies) were transfected by X-tremeGENE DNA transfection reagent (Roche). A day later, luciferase signals were quantitated by Dual-LuciferaseReporter Assay based on manufacturer’s protocol (Promega). LNA Ctrl (control) and LNA miR-122 were purchased from Exiqon and were transfected by X-tremeGEME DNA transfection reagent in PLC/PRF/5 cells based upon manufacturer’s protocol.

### Real-time quantitative PCR

Trizol Reagent (Life Technologies) was used to extract total RNA. cDNA synthesis was performed by reverse transcription kit (Life Technologies). SYBR Green PCR Master Mix (Life Technologies) and primers specific to PKL, PKR, PKM1, and PKM2 (S1 Table) were used for real-time quantitative PCR (RT-qPCR).

### Clinicopathological correlation and patients’ survival analysis

The clinicopathological features of HCC patients were analyzed by pathologist (Irene Oi-Lin Ng), as we previously described [11]. The parameters consisted of tumor size, venous invasion, tumor microsatellite formation, tumor encapsulation, cellular differentiation by Edmondson grading, direct liver invasion, background liver disease, and pTNM tumor stage. These features were correlated with PKM2 mRNA expression by Mann Whitney test. Tumor recurrence rate was calculated from the date of resection to the date when recurrence was first detected or, in the absence of tumor, to one year after resection. Overall survival was calculated from the date of resection to the date of death or five years after resection. The prognostic significance of PKM2 overexpression (T/NT2 fold) was determined by the Kaplan-Meier method followed by the log-rank test. All statistical tests were performed with SPSS20.0 and considered significant when the *P*-values were less than 0.05.

### Animal studies

All protocols for animal experiments were approved by the Committee on the Use of Live Animals in Teaching and Research, the University of Hong Kong and followed under the Animals (Control of Experiments) Ordinance of Hong Kong. For subcutaneous injections in nude mice, 1×10^6^ MHCC-97L or SMMC-7721 cells were injected into the right flanks of 6–8 weeks old male BALB/c NUDE mice. Three dimensions of tumors were measured by caliper every week. Tumor volume was calculated by the following equation: width (mm) × length (mm) × depth (mm)×0.52 [12], [13]. Tumor volume of each group was plotted against time and compared to the corresponding controls. Mice were sacrificed 4 weeks after the subcutaneous injection. Tumors were harvested and weighed. For orthotopic liver tumor implantation in nude mice, 1×10^6^ luciferase-labeled MHCC-97L cells were injected into the left lobes of the livers of 6–8 week-old nude mice. Six weeks after implantation, mice were administered with 100 mg/kg D-luciferin 5 minutes before bioluminescent imaging (IVIS^TM^100 Imaging System, Xenogen, Hopkinton, MA). Lungs were harvested for *ex-vivo* imaging and histological analysis. Livers were harvested and three dimensions of tumors were measured for tumor volume calculation.

### Lactate, glucose uptake, reactive oxygen species (ROS), and NADPH measurement

Lactate secreted by cells was quantitated by Lactate Colorimetric Assay Kit (BioVision Inc Milpitas, CA). Lactate accumulation was calculated based on the formula: lactate level/number of cells. Glucose uptake by cells was quantitated by measuring the initial and final glucose content in the conditioned medium by Glucose Colorimetric Assay Kit (BioVision Inc). Glucose uptake rate was calculated based on the formula: ([initial glucose]-[final glucose])/time/number of cells. To confirm the glucose colorimetric assay, cells were stained with glucose analog 2-(N- (7-Nitrobenz-2-oxa- 1,3-diazol-4-yl) Amin)-2-Deoxyglucose (2-NBDG) (Life Technologies) followed by flow cytometry analysis. For ROS measurement, trypsinized cells were washed with PBS and stained with general ROS indicator chloromethyl-2′, 7′-dichlorodihydrofluorescein diacetate (CM-H_2_DCFDA) (Life Technologies) and analyzed by flow cytometer. Data from flow cytometry studies were analyzed by FlowJo software. NADPH levels in cells were measured with NAPDH Quantification Colorimetric Kit (BioVision Inc).

### Antibodies, HCC tissue microarray sections, immunohistochemistry and histology

PKM1 (Sigma), phospho-PKM2 (Tyr105) (Cell Signaling Technology, Danvers, MA), PKM2 (Cell Signaling Technology), PKL (Abcam), β actin (Sigma) were used for Western Blotting. Paraffin sections were washed with xylene and rinsed with ethanol. Antigens were retrieved in EDTA buffer by boiling. Tissue microarray or individual clinical tissue sections were stained with PKM2 and PKL antibodies and slightly stained with hematoxylin. Mouse tissue sections were stained with hematoxylin and eosin (H&E) for histological analysis.

## Results

### Expression of PK isoforms in human HCC

**Figure 1:**
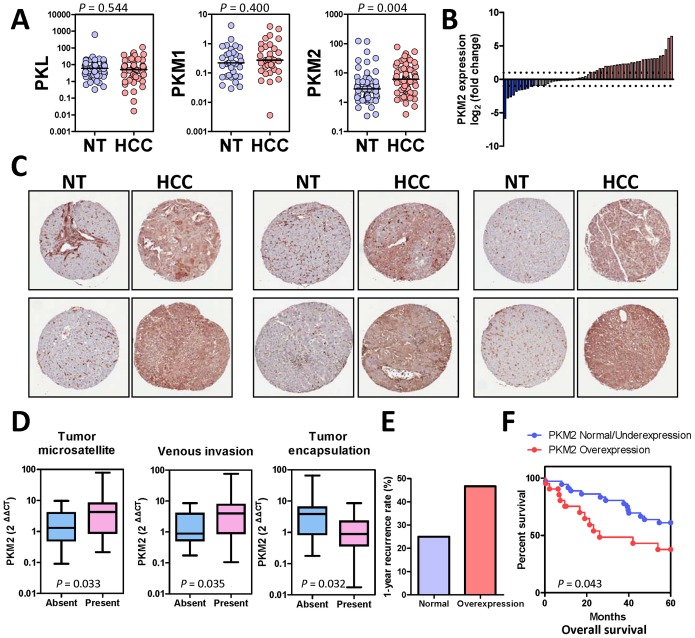
PKM2 expression in human HCC. (A) mRNA expression of PKL, PKM1, and PKM2 in HCC and NT tissues. Values = 2^ΔΔCT^, ΔΔCT = (CT_PK_ – CT_HPRT_) of HCC - (CT_PK_– CT_HPRT_) of NT. *P* values, Wilcoxin signed rank test (B) Waterfall plot shows that, at the mRNA level, PKM2 was up-regulated (HCC/NT2 folds) in 29/60 (48.33%) human HCC samples. (C) Representative pictures of IHC staining with antibody against PKM2 in HCC tissue microarray. PKM2 protein was drastically up-regulated in human HCCs as compared to the paired NT tissues. (D) Mann Whitney test showed that PKM2 over-expression was associated with multiple aggressive clinicopathological features in HCC including the presence of tumor microsatellites, presence of venous invasion, and absence of tumor encapsulation. (E) Over-expression of PKM2 in human HCC was associated with poor prognosis. HCC patients were categorized into two groups: PKM2 over-expression and PKM2 normal/under-expression. PKM2 was considered to be over-expressed when HCC/NT2 folds and was considered to be normal/under-expressed otherwise. HCC patients with PKM2 over-expression had a higher 1-year tumor recurrence rate after surgical resection than HCC patients without PKM2 over-expression, 46.667% Vs 25%. (F) Patients with PKM2 over-expression had lower 5-year overall survival rates after surgical resection. *P* values were calculated by Kaplan-Meir log rank test.

To study the expression of different forms of the PK family, we designed RT-qPCR primers that were specific to individual isoforms and assessed their expression in 60 cases of human HCC samples and their corresponding non-tumorous liver tissues (NT). Interestingly, we found that only PKM2 was over-expressed in the human HCCs while PKM1 and PKL expression levels remained unchanged (Fig. 1A). PKR expression was undetectable in both HCC and NT tissues (data not shown). Notably, PKL was the predominant form in the NT livers but its expression was unchanged in HCC tissues, implying that PKL might contribute to the normal metabolic functions in the livers while PKM2 might contribute to metabolic functions in HCC. At the mRNA level, PKM2 was up-regulated by at least two-fold in 29 (48.33%) of the 60 human HCC samples (Fig. 1B). To further examine the PKM2 protein expression, we performed immunohistochemistry (IHC) study with an antibody against PKM2 on the tissue microarray slides containing tissue cores from 109 HCCs and their corresponding NT livers. We found that PKM2 was over-expressed in 68.8% (75/109) of the HCC patients (Fig. 1C). In line with mRNA expression study, PKL protein was not altered in human HCCs (S1 Fig.).

### Clinicopathological correlation and prognostication of PKM2 in human HCCs

Over-expression of PKM2 in HCC tumors was significantly associated with aggressive pathological features including tumor microsatellite formation (*P* = 0.033), venous invasion (*P* = 0.035), and absence of tumor encapsulation (*P* = 0.032) (Fig. 1D). More importantly, HCC patients with PKM2 over-expression have higher 1-year recurrence rate than HCC patients without PKM2 over-expression (46.667% versus 25%) (Fig. 1E). Furthermore, over-expression of PKM2 was associated with shorter overall survival (*P* = 0.043) in HCC patients (Fig. 1F).

### PKM2 and HCC metabolism

**Figure 2:**
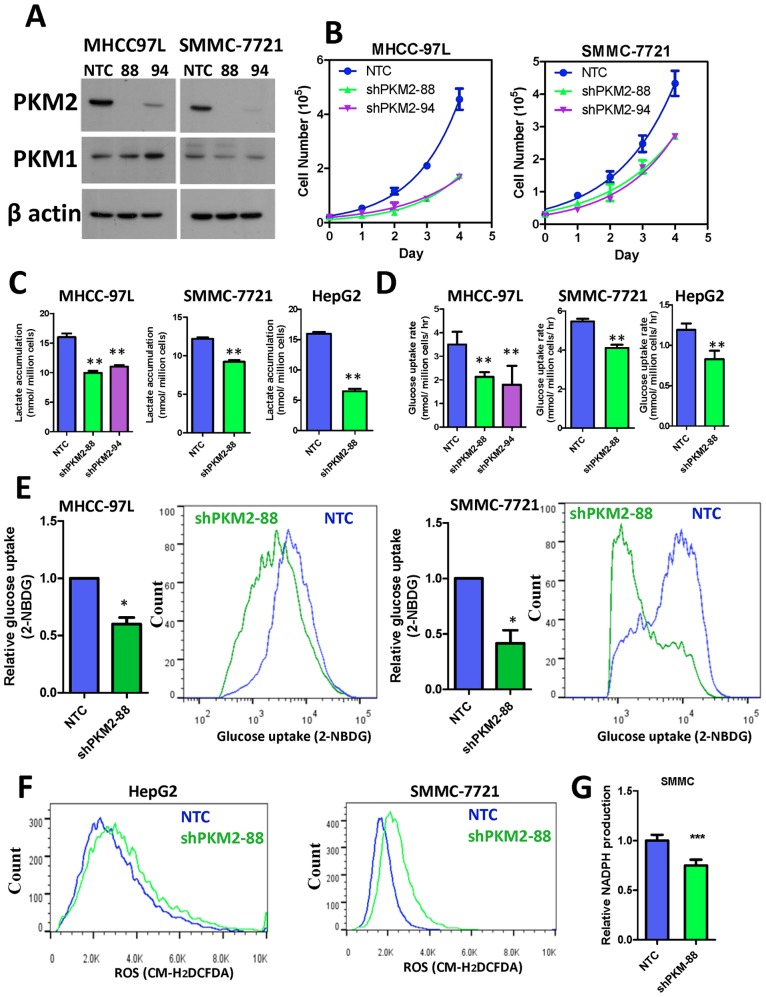
PKM2 promoted HCC growth *in* *vitro* through regulating aerobic glycolysis and ROS levels. (A) Two stable PKM2 knockdown clones were generated in MHCC-97L and SMMC-7721 cells. Expression of PKM2, PKM1, and β actin were evaluated by Western Blots. (B) Knockdown of PKM2 by two independent sequences consistently reduced HCC cell proliferation rate by cell counting. (C) Knockdown of PKM2 reduced lactate accumulation in multiple HCC cell lines. (D) Colorimetric assay showed that knockdown of PKM2 reduced the glucose consumption rate of multiple HCC cell lines. (E) Glucose uptake in HCC cells was confirmed with 2-NBDG staining. (F) Knockdown of PKM2 increased ROS accumulation in multiple HCC cells. (G) Knockdown of PKM2 decreased NADPH level in SMMC-7721 cells. Values were normalized to NTC of the according cell lines. *P<0.05, **P<0.01, **P<0.001 Student’s *t* test (n≧3).

To study the functional implications of PKM2, we employed short-hairpin RNA (shRNA) approach to stably knockdown PKM2 in multiple HCC cell lines such as SMMC-7721 and MHCC-97L, that expressed a high level of PKM2 (Fig. 2A). Theoretically, these shRNA sequences could not distinguish PKM2 and PKM1 isoforms. However, we found these sequences only affected PKM2 but not PKM1. This may be due to the basically low expression of PKM1 in these HCC cell lines. PKM1 was only barely detected at the same exposure time when PKM2 was already easily detected (S2A Fig.). The mRNA expression values of PKM2 and PKM1 in human HCC samples and cell lines also reflected that the expression of PKM1 was much lower than PKM2 in HCC (Fig. 1A, S2B Fig.). Different shRNA knockdown sequences of PKM2 but not PKL consistently suppressed HCC cell proliferation when compared to the non-target control (NTC) shRNA (Fig. 2B and S3 Fig.). To investigate whether PKM2 induced the Warburg effect in HCC cells, we assessed lactate accumulation and glucose uptake rate in the –NTC and –shPKM2 HCC cells under aerobic conditions. Knockdown of PKM2 consistently suppressed lactate accumulation (Fig. 2C) and glucose uptake rate (using both colorimetric assay and 2-NBDG staining) in multiple HCC cell lines (Fig. 2D and 2E). In addition, knockdown of PKM2 substantially induced intracellular ROS level in multiple HCC cell lines (Fig. 2F). Increase of ROS might be caused by a reduction of NADPH. In line with our postulation, we found that knockdown of PKM2 decreased the NADPH level in SMMC-7721 cells (Fig. 2G).

### Knockdown of PKM2 suppressed tumor growth and metastasis in vivo

**Figure 3:**
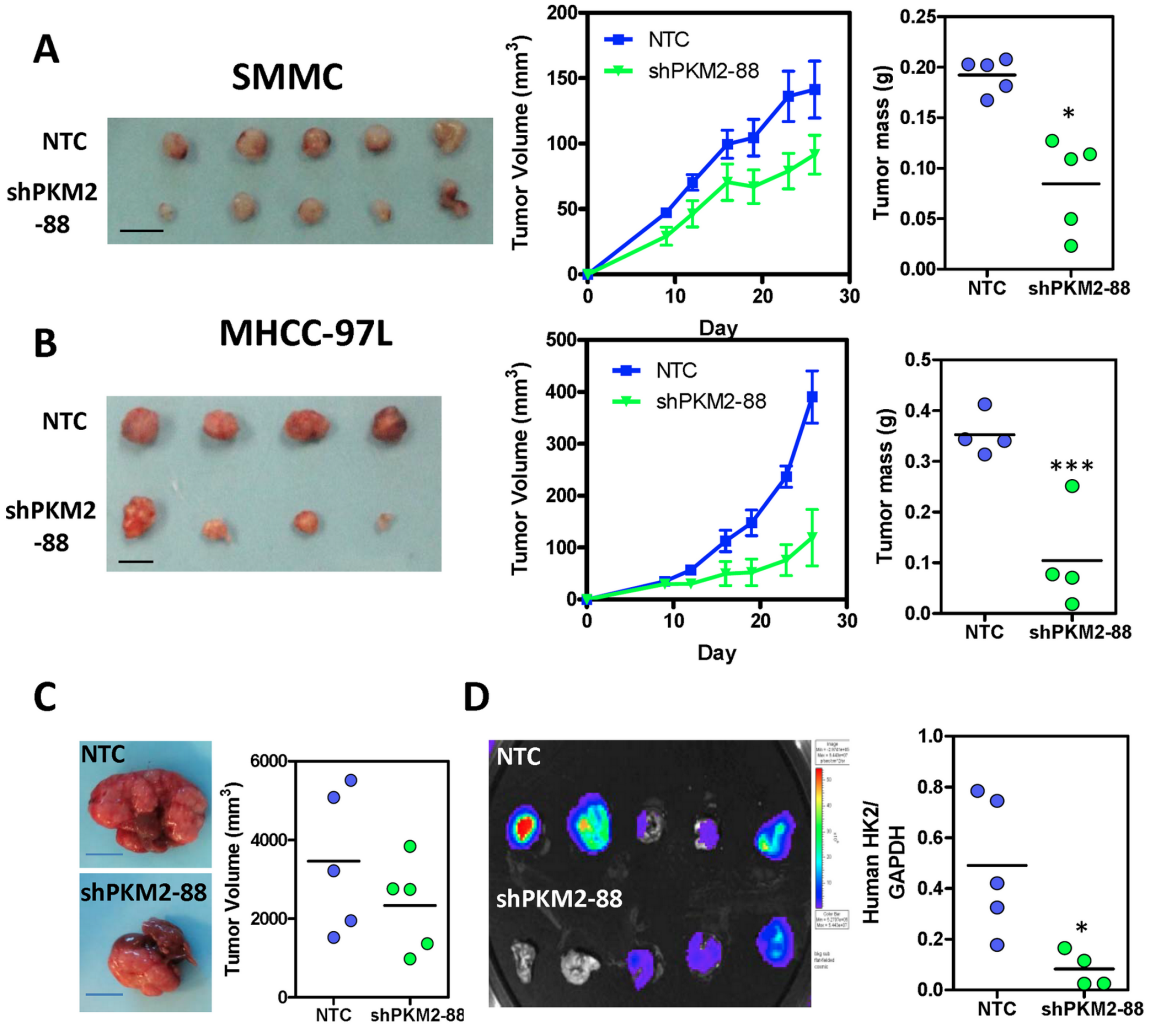
PKM2 promoted HCC growth *in* *vivo*. (A) Left: Subcutaneous tumors derived from SMMC-NTC, -shPKM2 cells. Middle: volumes (mm^3^) of SMMC-NTC and -shPKM2 tumors were measured and plotted against time. Right: mass (g) of SMMC-NTC, -shPKM2 tumors were measured at the end of the experiment. (**P<0.01, Student’s t test) (B) Left: subcutaneous tumors derived from MHCC-97L-NTC and -shPKM2 cells. Middle: volumes (mm^3^) of MHCC-97L-NTC and -shPKM2 tumors were measured and plotted against time. Right: mass (g) of MHCC-97L-NTC and -shPKM2 tumors were measured at the end of the experiment. (C) Left: orthotopic tumors derived from MHCC-97L-NTC and -shPKM2 cells. Right: Tumor volume was measured at the end of the experiment. (D) Left: bioluminescent signals of the lung tissues in the mice orthotopically implanted with luciferase labeled-MHCC-97L-NTC and -shPKM2 cells. Right: mRNA expression of human hexokinase 2 (HK2) in lung tissues of mice orthotopically implanted with luciferase-labeled MHCC-97L-NTC and -shPKM2 cells. Values were normalized to mouse GAPDH. *P<0.05, **P<0.01,***P<0.001, Student’s t test. Scale: 1 cm.

To evaluate the effect of PKM2 in HCC growth *in* *vivo*, we subcutaneously injected SMMC-7721- and MHCC-97L-NTC and -shPKM2 cells into nude mice and found that knockdown of PKM2 drastically hampered tumor growth (Fig. 3A and 3B). Furthermore, to better mimic the tumor microenvironment of human HCC, we orthotopically injected luciferase-labeled MHCC-97L-NTC and -shPKM2 cells into the left lobes of the livers of nude mice. Knockdown of PKM2 not only suppressed tumor growth in the livers (Fig. 3C) but also suppressed the growth of metastatic lesions found in the lungs as shown by the Xenogen imaging and expression of human genes *HK2* in the mouse lung tissues (5/5 mice in NTC vs 3/5 mice in shPKM2) (Fig. 3D).

### PKM2 regulation in human HCC

**Figure 4:**
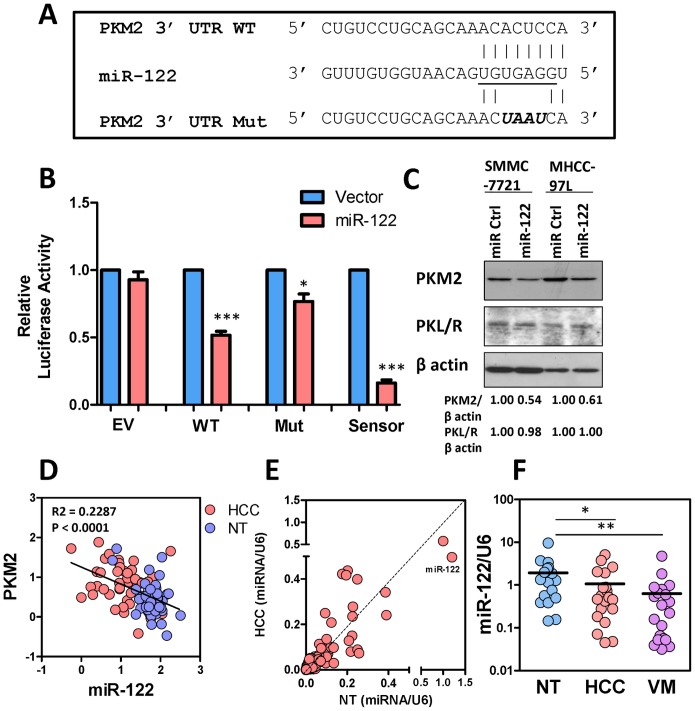
MiR-122 targeted PKM2 and suppressed PKM2 expression. (A) Seed sequence of miR-122 in the 3UTR of PKM2 was underlined. WT and mutated sequences of PKM2 were inserted into pmiRGLO luciferase vector. (B) Re-expression of miR-122 suppressed luciferase activity of the WT but not the Mut 3UTR of PKM2. EV and sensor sequences served as negative and positive controls, respectively. (C) Re-expression of miR-122 in SMMC-7221 and MHCC-97L cells suppressed PKM2 but not PKL/R expression. (D) Linear regression model demonstrated that PKM2 mRNA expression was inversely correlated with miR-122 expression in HCC (pink) and NT liver tissues (blue). (E) Expression levels of 667 miRNA species in HCC and NT were plotted. Each dot represents one individual miRNA. MiR-122 is the most abundant miRNA in NT liver. (F) MiR-122 expression in NT, HCC, and venous metastases (VM). Data were retrieved from low density microarray in which expressions of 667 miRNA species were compared between NT, HCC, and VM. **B**, *P<0.05, ** *P*<0.01, *** *P*<0.001, Student’s t test (n≧3). **E**, *P<0.05, **P<0.01, Wilcoxon signed rank test.

Next, we investigated the mechanism driving PKM2 up-regulation in HCC. By *in silico* analysis with the algorithm Target Scan 5.2, we studied the 3UTR of PKM2 and found that miR-122 could potentially interact with the 3UTR of PKM2. MiR-122 theoretically could interact with the 3UTR of both PKM1 and PKM2. However, as HCC cells expressed high level of PKM2 but not PKM1 (Fig. 1A), the functional effects driven by miR-122 should mainly be contributed by PKM2. To validate the interaction between miR-122 and PKM2, we cloned the wild-type (WT) and mutant (Mut) 3UTRs of PKM2 into the pmirGLO luciferase reporter vector (Fig. 4A). We also cloned the sensor sequence which was completely complementary to miR-122 as a positive control. Luciferase reporter assay showed that miR-122 suppressed the WT 3UTR and effect was reduced in the Mut 3UTR of PKM2 (Fig. 4B). To assess whether PKM2 expression was affected by miR-122, we transiently transfected miR-122 precursors into HCC cell lines and found that miR-122 suppressed the protein expression of PKM2 but not PKL, the predominant form in normal livers (Fig. 4C). Interestingly, miR-122 precursors did not affect the phosphorylation of PKM2 at Tyrosine 105, indicating that miR-122 only regulated the expression but not the activity of PKM2 (S4 Fig.). Importantly, linear regression model showed that miR-122 expression inversely correlated with PKM2 expression in our cohort of HCC and NT liver tissues (Fig. 4D).

### MiR-122 in human HCCs

**Figure 5:**
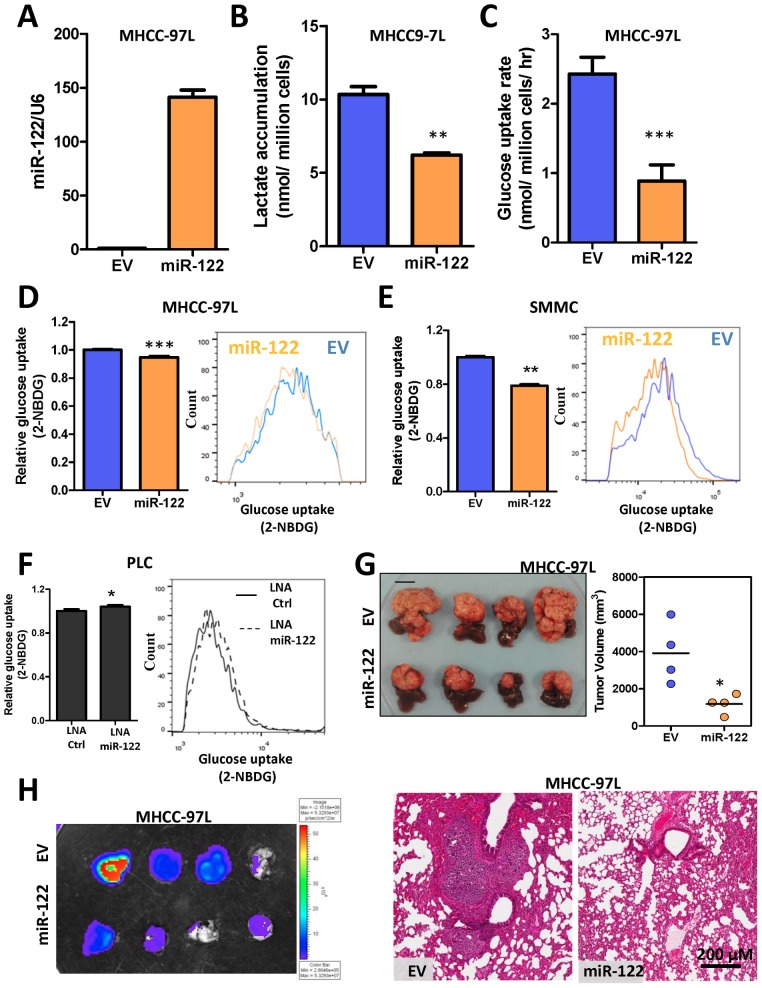
Re-expression of miR-122 suppressed HCC growth through modulating aerobic glycolysis. (A) miR-122 expression in MHCC-97L cells stably expressing miR-122 precursors. MiR-122 expression was normalized to U6 expression and to empty vector (EV) control. (B) Lactate accumulation was reduced in miR-122 over-expressing MHCC-97L cells. (C) Glucose uptake rate was reduced in miR-122 over-expressing MHCC-97L cells. (D) Glucose uptake in MHCC-97L-EV and –miR-122 cells was confirmed with 2-NBDG staining. (E) Glucose uptake in SMMC-EV and –miR-122 cells was confirmed with 2-NBDG staining. (F) Glucose uptake in PLC/PRF/5 cells transfected with LNA-Ctrl and LNA-miR-122. (G) Left: Orthotopic tumors derived from MHCC-97L-EV and -miR-122 subclones. Right: Tumor volume was measured at the end of the experiment. (H) Bioluminescence (left) and H&E staining (right) in lung tissues from mice implanted with MHCC-97L-EV and –miR-122 subclones. *P<0.05, **P<0.01, *** *P*<0.001, Student’s t test or paired t test. Scale: 1 cm.

To further interrogate the roles of miR-122 in human HCC, we looked into our previous miRNA array study which compared the expression of>667 microRNA species in tumor and NT specimens in 20 HCC patients. Consistent with other reports, miR-122 was one of the most abundantly expressed miRNAs in the human liver (Fig. 4E). The abundance of miR-122 in the human liver implies that miR-122 might play an important role in human liver. Moreover, we found that miR-122 was significantly under-expressed in human HCC tissues as compared to NT livers and was further under-expressed in venous metastases (VM) (Fig. 4F). As we have shown earlier in this study that PKM2 promoted the Warburg effect in HCC, we studied the metabolic roles of miR-122 in HCC cell lines. We generated MHCC-97L and SMMC-7721 cells that stably expressed miR-122 (Fig. 5A) and found that miR-122 profoundly reduced the PKM2 expression (S5 Fig.) lactate accumulation (Fig. 5B) and glucose uptake rate (Fig. 5C–E) in MHCC-97L cells. Conversely, inhibition of miR-122 in HCC cell line with high expression of miR-122, PLC/PRF/5, by LNA enhanced glucose uptake rate (Fig. 5F). More importantly, re-expression of miR-122 in MHCC-97L significantly impeded tumor growth and attenuated lung metastasis (Fig. 5G–H).

## Discussion

Our study has provided evidence that PKM2 was over-expressed in human HCCs. Our finding further supports the general observation that PKM2 is the isoform particularly expressed in tumors. Furthermore, we found that PKM2 over-expression was closely associated with aggressive pathological HCC features and poor survival rates. Our findings, together with others, suggest that PKM2 over-expression confers advantage to cancer cell growth. Conversely, a number of studies have shown that reduction of PKM2 enzymatic activity promotes tumor growth by influencing the generation of anabolic intermediates [14]–[16]. Phosphotyrosine phosphorylation, reactive oxygen species, and lysine acetylation have been shown to modulate the activity of PKM2 [14]–[16]. Small-molecule activators of PKM2 also suppressed tumor growth [17]. Although it remains intriguing why a lowered PKM2 activity would benefit tumor growth while PKM2 is the predominant form found in cancers, all these studies have clearly indicated that deregulation of PKM2 is pivotal to tumor growth. Of note, these studies only compared the oncogenic activities of PKM2 and PKM1 as most cancers do not express PKL/R isoforms. In the context of the liver, PKL is the most dominant isoform. Our data clearly showed that knockdown of PKM2 but not PKL in HCC cells suppressed HCC proliferation. This finding seems to be in contradiction with a recent publication showing that mice with specific knockout of PKM2 are prone to breast cancer development [18]. The discrepancy between this study and others can be attributed by the compensatory effect of PKL in HCC and different animal experimental models. Therefore, how the activities of PKL, PKM1, and PKM2 are maintained along hepatocarcinogenesis to maximize HCC growth will be the next important question to be addressed in the future. Although we believe that specific knockdown or inhibition of PKM1 would only have minimal effect in HCC growth due to the abundance of other pyruvate kinase isoforms in HCC, the roles of PKM1 in HCC merit further investigation.

MicroRNAs (miRNAs) are a class of endogenous non-coding small RNAs that interact with the 3 untranslated region (UTR) of their target genes, thereby regulating their expression [19]. Our study showed that re-expression of miR-122 suppressed PKM2 in HCC cell lines; therefore, under-expression of miR-122 may contribute to preferential expression of PKM2 in human HCC. On the other hand, in normal liver, miR-122 is highly expressed and suppresses PKM2, making PKL the most abundant PK isoform. In addition to miR-122 we reported in our current study, a number of miRNAs have been shown to regulate genes involved in the glucose metabolism, thereby fine-tuning cancer metabolism. MiR-133 was shown to suppress glucose transporter, GLUT4, through Kruppel-like factor 5 (KLF5) in cardiomyocytes [20]. Insulin-stimulated miR-93 directly interacted with the 3UTR of GLUT4 and subsequently suppressed GLUT4 expression in adipose tissues [21]. MiR-143 and miR-138 down-regulated glycolytic enzymes, hexokinase 2 (HK2) and HK1, respectively, in head and neck carcinoma [19]. A hypoxia-induced miRNA, miR-210, interacted with the 3UTR of iron-sulfur cluster assembly proteins (ISCU1/2) and suppressed expression of ISCU1/2 [22]. As ISCU1/2 participate in the electron transport through complex I, III, and aconitase and produce ROS during oxidative phosphorylation, miR-210 is critical to the optimization of the redox reactions under a hypoxic environment [22]. Many of these miRNAs, like miR-122, have been reported to be deregulated in various cancer tissues (please see review for details [23], indicating the importance of miRNAs in cancer metabolism.

MiR-122 is the most dominant miRNA in normal hepatocytes and it is important for the maintenance of normal liver functions. MiR-122 was shown to regulate a network of genes that are responsible for lipid metabolism [24], mitochondrial metabolism [25], and differentiation [26]. Mouse with germline deletion of miR-122 developed steatohepatitis, fibrosis, and HCC [27]. Interestingly, in a genome-wide expression profiling in 96 pairs of HCC and NT liver tissues which analyzed the coordinate expression of mRNAs and miRNAs, miR-122 under-expression in HCC was found to be significantly associated with the genes involved in mitochondrial functions, fatty-acid metabolism, and amino-acid metabolism [25]. However, PKM2 has not been mentioned in this comprehensive bioinformatics study [25]. In another study which compared the gene expression profiles of human embryonic stem cells (hESCs), human primary hepatocytes (hPHs), and HCCs, miR-122 was found to be significantly under-expressed in hESCs and hPHs [28]. In this study, miR-122 was shown to suppress stem cell-renewal and HCC proliferation through down-regulating PKM2 [28]. Although the relationship of miR-122 and PKM2 has been briefly revealed [28], the defined roles of miR-122 in HCC metabolism especially in the aspect of glucose metabolism have not been clearly elucidated. Our current study has unequivocally revealed the precise mechanisms by which miR-122 regulated the PK isoform switching in HCC, the clinical relevance of PKM2, and the clinical association of PKM2 and miR-122.

At the time of manuscript submission, we noticed a report by Liu *et* *al*. that shares similar findings with our current study [29]. Although Liu *et* *al*. started from the angle of miR-122 and discovered PKM2 as its target while our current study started from the angle of different PK isoforms and discovered miR-122 as the regulator of PKM2, two studies converged to an important conclusion. While our data echo Liu *et* *al*.’s *in* *vitro* findings, we additionally demonstrated that PKM2 and miR-122 were critical to HCC growth *in* *vivo* using both subcutaneous and orthotopic implantation models. Furthermore, we showed that PKL was the dominant isoform in normal liver while PKM2 was overexpressed in HCC. Our *in* *vivo* data and findings on different PK isoforms will be particularly important to preclinical studies which involve the design of therapeutic strategies that specifically target PKM2. The clinicopathological findings from the two studies are consistent although different primers were employed, reinforcing the important diagnostic and prognostic roles of PKM2 in HCC. Intriguingly, we showed that overexpression was associated with higher recurrence rate. Of note, in addition to the effect of PKM2 in lactate production that both studies reported, we showed that knockdown of PKM2 suppressed the glucose uptake rate and increased the oxidative stress in HCC cells. This information is especially important to the understanding of the complex metabolic machinery of HCC. Our data suggested that PKM2 may channel glucose intermediates (glucose-6-phosphate) to the pentose phosphate shunt which produces NADPH to reduce the level of ROS in the cells, therefore further conferring a growth advantage to HCC cells. Taken together, Liu *et* *al.*’s and our current studies contain complementary information supporting the important metabolic roles of miR-122/PKM2 in HCC development.

## Supporting Information

S1 Fig **PKL expression in human HCC and NT tissues.** Representative IHC pictures of HCC and NT tissues stained with PKL antibody.
(TIF)

S2 Fig **Expression of PKM2 and PKM1 in HCC cell lines.** (A) Protein lystates from MHCC-97L- and SMMC-NTC, shPKM2-88, shPKM2-94 were probed with PKM2, phosphoPKM2 (Tyrosine 105), PKM1, β actin antibodies at various exposure. (B) mRNA expression levels of PKM1 and PKM2 in MHCC-97L and SMMC-7721 cells (ΔCT = (CT_PK_ – CT_18S_)).
(TIF)

S3 Fig **PKL did not affect HCC cell proliferation**. (A) PKL mRNA expression in SMMC-7721 cells stably expressing 2 independent shRNA sequences (39 and 94) targeting PKL. (B) Knockdown of PKL only mildly reduced HCC cell proliferation rate.
(TIF)

S4 Fig **MiR-122 reduced expression but not the activity of PKM2.** Protein lysates were from SMMC-7721 cells that expressed miR-122 and miR-122 control precursors were probed with phosphoPKM2 (Tyrosine 105), PKM2, and β actin antibodies. Band intensities were quantitated by Image U and values were normalized with the corresponding NTC.
(TIF)

S5 Fig **PKM2 mRNA expression in MHCC-97L and SMMC-7721 cells that stably expressed EV or miR-122.** Values were normalized with 18S and their corresponding EV.
(TIF)

S1 Table **Primer sequences used in RT-qPCR study.** Sequences of forward and reverse primers used for RT-qPCR study for the quantitation of the expression levels of human PKM1, human PKM2, human PKL, human PKR, human HPRT, human HK2, and mouse GAPDH.
(DOCX)
