# Supplementary figures and images for: Switching of Pyruvate Kinase Isoform L to M2 Promotes Metabolic Reprogramming in Hepatocarcinogenesis
Source: PLoS One. 2014 Dec 26;9(12):e115036. doi: 10.1371/journal.pone.0115036 (PMC4277479; doi:10.1371/journal.pone.0115036)

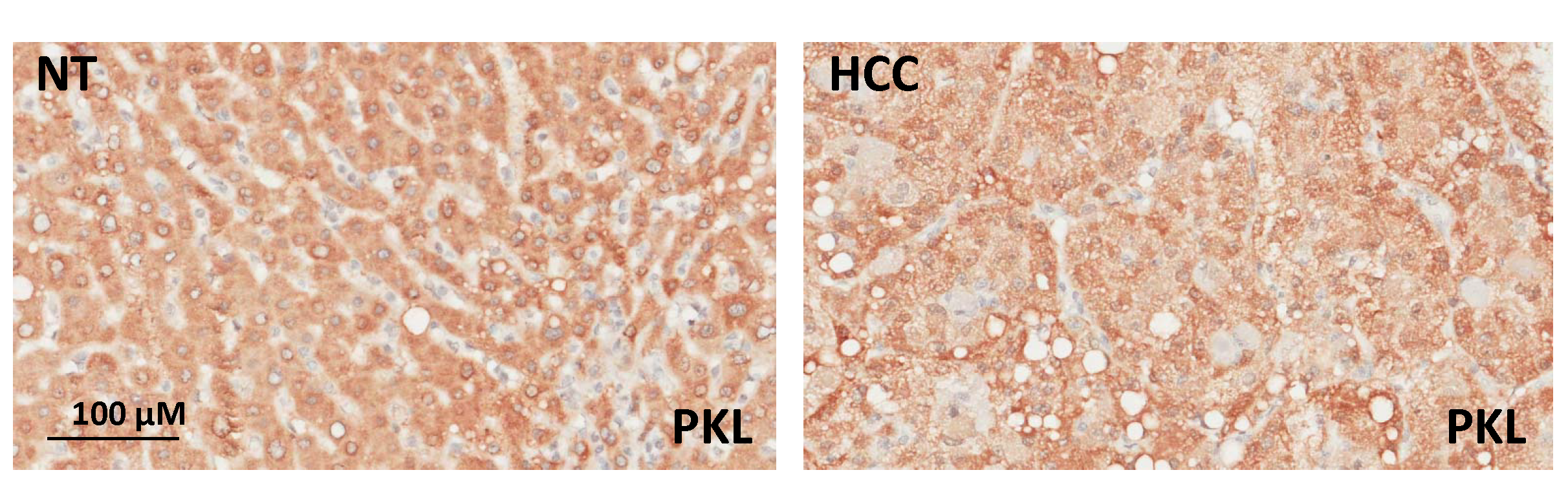

Supplement: S1 Fig — PKL expression in human HCC and NT tissues. Representative IHC pictures of HCC and NT tissues stained with PKL antibody. (TIF) [file pone.0115036.s001.tif]

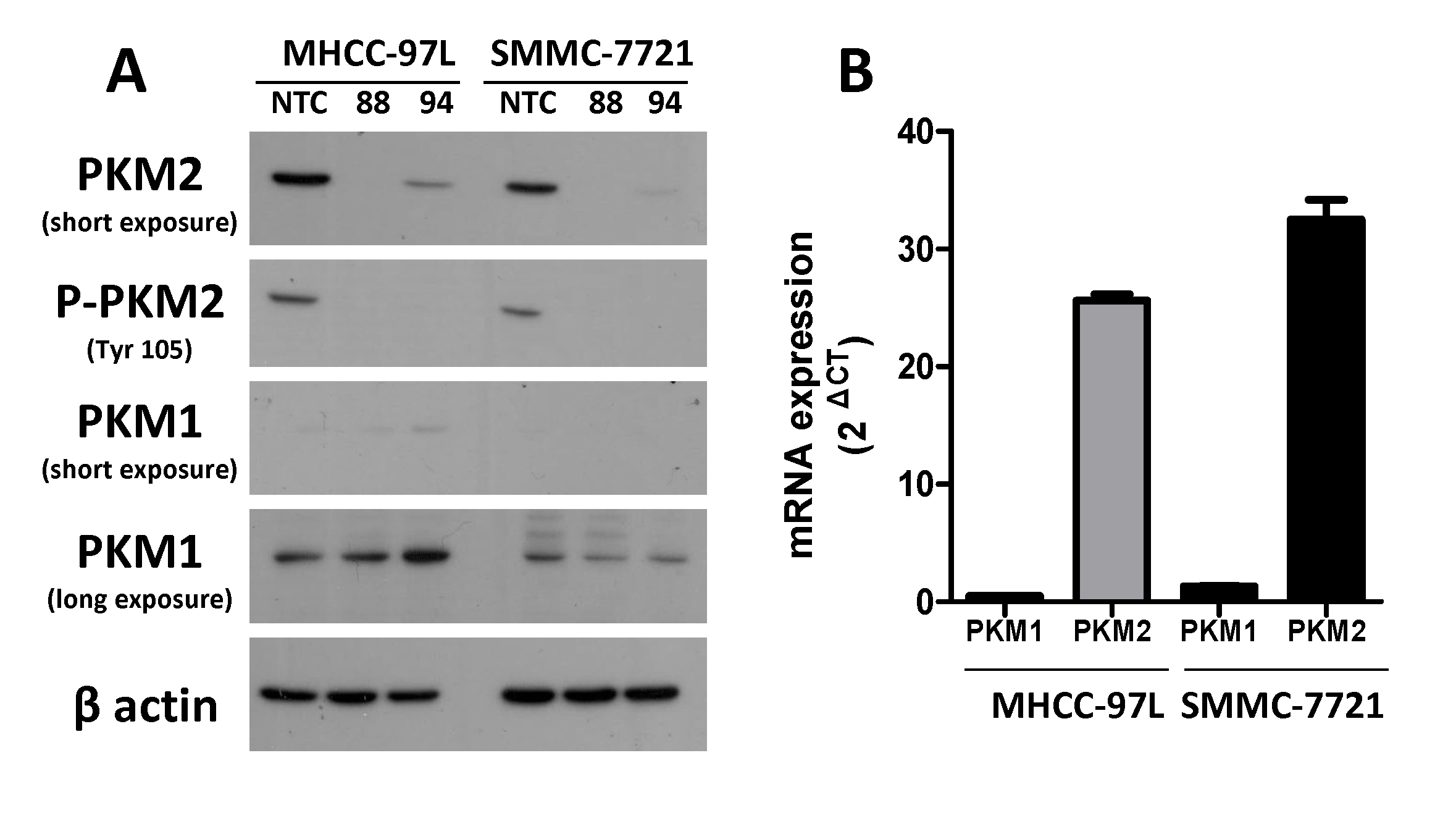

Supplement: S2 Fig — Expression of PKM2 and PKM1 in HCC cell lines. (A) Protein lystates from MHCC-97L- and SMMC-NTC, shPKM2-88, shPKM2-94 were probed with PKM2, phosphoPKM2 (Tyrosine 105), PKM1, β actin antibodies at various exposure. (B) mRNA expression levels of PKM1 and PKM2 in MHCC-97L and SMMC-7721 cells (ΔCT = (CTPK – CT18S)). (TIF) [file pone.0115036.s002.tif]

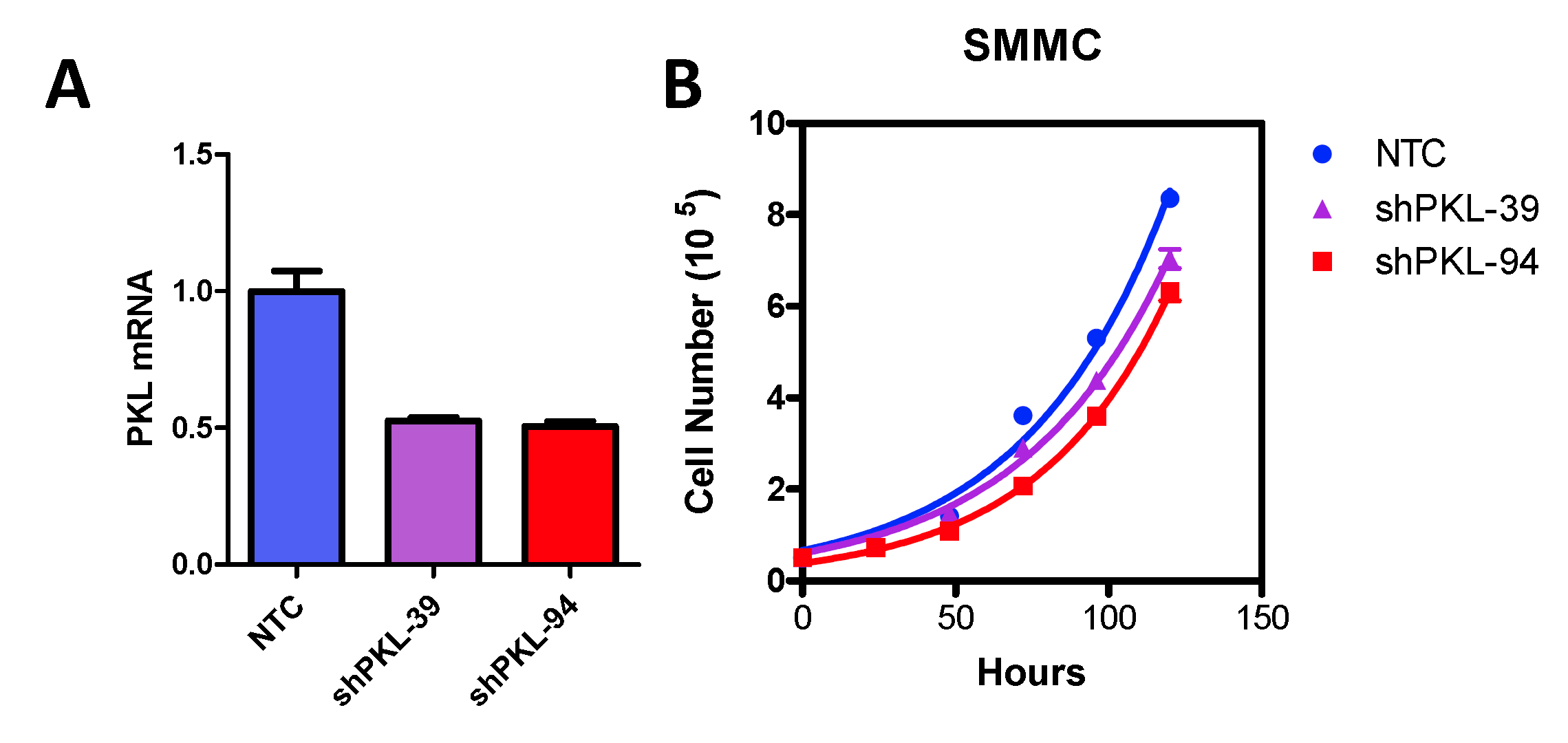

Supplement: S3 Fig — PKL did not affect HCC cell proliferation. (A) PKL mRNA expression in SMMC-7721 cells stably expressing 2 independent shRNA sequences (39 and 94) targeting PKL. (B) Knockdown of PKL only mildly reduced HCC cell proliferation rate. (TIF) [file pone.0115036.s003.tif]

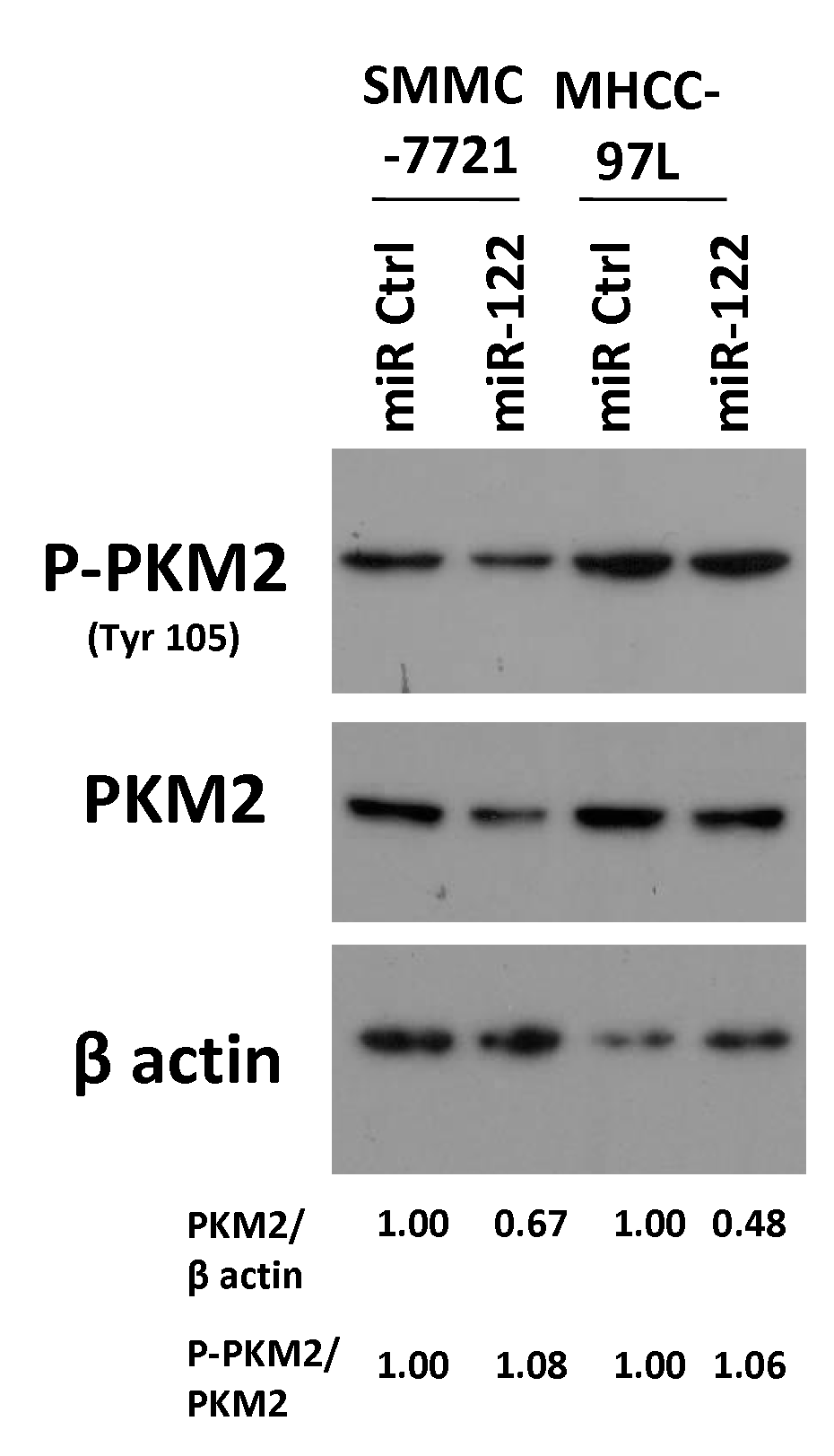

Supplement: S4 Fig — MiR-122 reduced expression but not the activity of PKM2. Protein lysates were from SMMC-7721 cells that expressed miR-122 and miR-122 control precursors were probed with phosphoPKM2 (Tyrosine 105), PKM2, and β actin antibodies. Band intensities were quantitated by Image U and values were normalized with the corresponding NTC. (TIF) [file pone.0115036.s004.tif]

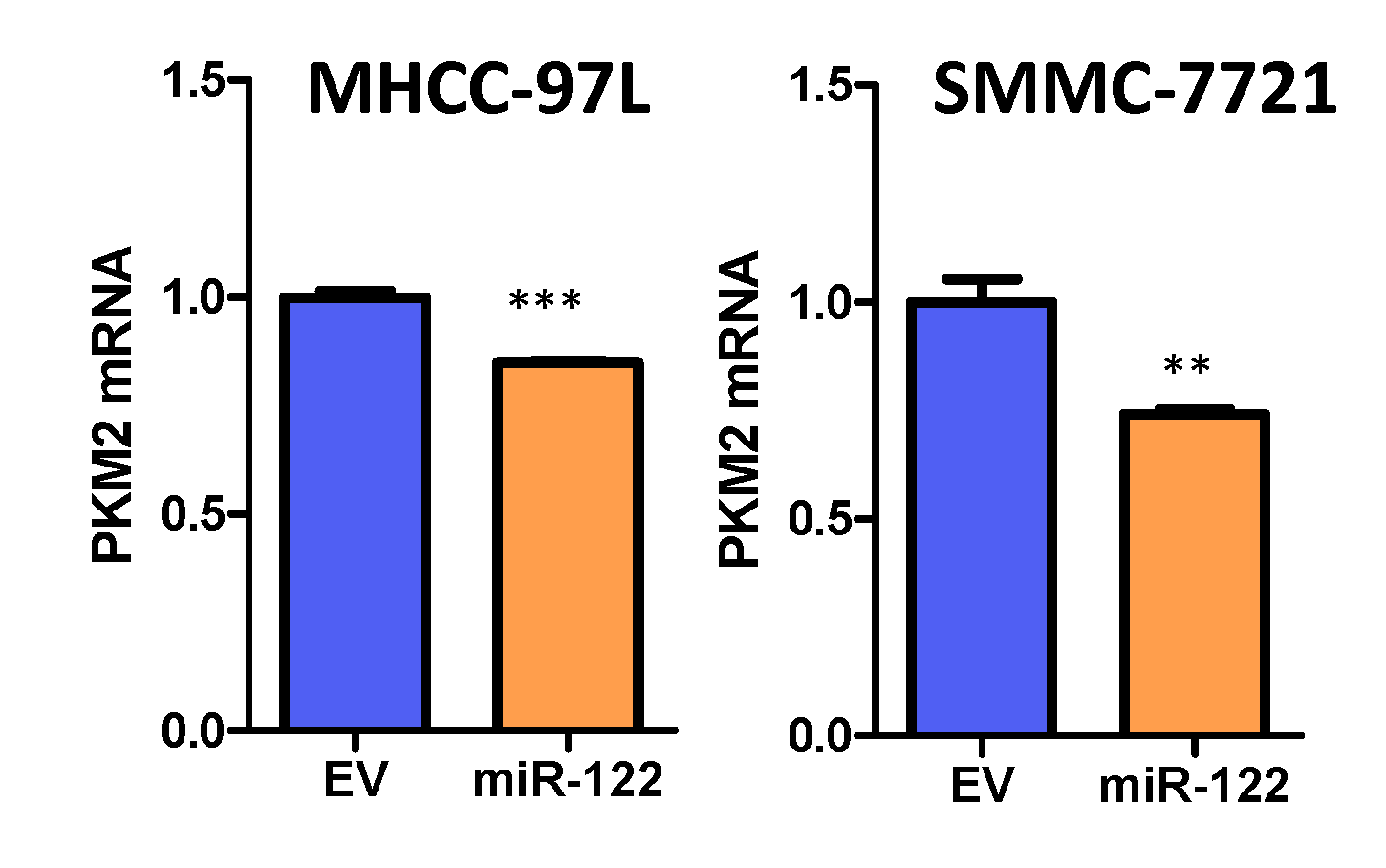

Supplement: S5 Fig — PKM2 mRNA expression in MHCC-97L and SMMC-7721 cells that stably expressed EV or miR-122. Values were normalized with 18S and their corresponding EV. (TIF) [file pone.0115036.s005.tif]
