# Supplementary material for: Switching of Pyruvate Kinase Isoform L to M2 Promotes Metabolic Reprogramming in Hepatocarcinogenesis
Source: PLoS One. 2014 Dec 26;9(12):e115036. doi: 10.1371/journal.pone.0115036 (PMC4277479; doi:10.1371/journal.pone.0115036)
Supplement: S1 Table — Primer sequences used in RT-qPCR study. Sequences of forward and reverse primers used for RT-qPCR study for the quantitation of the expression levels of human PKM1, human PKM2, human PKL, human PKR, human HPRT, human HK2, and mouse GAPDH. (DOCX) [file pone.0115036.s006.docx]

**Table S1.** **Primer sequences used in RT-qPCR study.**

| **Genes** | **Sequences** |
| --- | --- |
| Human PKM1 | Forward: tcactccacagacctcatgg  Reverse: gaagatgccacggtacaggt |
| Human PKM2 | Forward: atcgtcctcaccaagtctgg  Reverse: gaagatgccacggtacaggt |
| Human PKL | Forward: gagagggagacccagagagg  Reverse: cagctcctgggtcagttgg |
| Human PKR | Forward: cttagcaaagtccatcctgatt  Reverse: cagctcctgggtcagttgg |
| Human HPRT | Forward: ctttgctgacctgctggatt  Reverse: ctgcattgttttcggagtgt |
| Human HK2 | Forward: ccagttcattcacatcatcag  Reverse: cttacacgaggtcacatagc |
| Mouse GAPDH | Forward: gccaaggtcatccatgacaac  Reverse: cttactccttggaggccatgt |
|  |  |
